# Evaluation of C-reactive protein and interleukin-6 in the peripheral blood of patients with chronic periodontitis

**DOI:** 10.4103/0972-124X.55840

**Published:** 2009

**Authors:** Dhruva Kumar Gani, Deepa Lakshmi, Rama Krishnan, Pamela Emmadi

**Affiliations:** *Sr. Lecturer, Department of Periodontics, Meenakshi Ammal Dental College, Chennai, India*; 1 *Professor, Department of Periodontics, Meenakshi Ammal Dental College, Chennai, India*

**Keywords:** Chemiluminescent immunoassay technique, C-reactive protein, interleukin-6, particle enhanced turbidimetric immunoassay technique

## Abstract

**Aims and Objectives:**

The aim of the present study was to investigate systemic levels of inflammatory markers of cardiovascular diseases like C-reactive protein and interleukin-6 in patients with chronic periodontitis, in comparison to periodontally healthy individuals.

**Materials and Methods:**

A total of 42 individuals, both males and females above the age of 30 years, were included in the study. Healthy controls (Group I, *n* = 14), chronic localized periodontitis (Group II, *n* = 14), and chronic generalized periodontitis (Group III, *n* = 14), all without any medical disorder, were recruited. Peripheral blood samples were taken and C-reactive protein (CRP) levels were estimated in the serum samples by using the Particle-Enhanced Turbidimetric Immunoassay (PETIA) technique. Serum samples of Interleukin-6 (IL-6) were assayed by using the Chemiluminescent Immunoassay (IMMULITE) technique.

**Results:**

When mean CRP levels were compared between the groups, group III showed statistical significance when compared to group I (*P* = 0.04). Group III had a higher median IL-6 level (6.35 pg/mL) than Group II (< 5.0 pg/mL) and group I (< 5.0 pg/mL). Differences in median values of IL-6 were not statistically significant in any group (*P* = 0.29).

**Conclusion:**

Periodontitis results in higher systemic levels of CRP and IL-6. These elevated inflammatory factors may increase inflammatory activity in atherosclerotic lesions and potentially increasing the risk for cardiovascular events.

## INTRODUCTION

One of the most common and often undiagnosed human diseases is periodontitis. This is a chronic infection of the supporting tissues of the teeth. It is estimated that about 15% of adults aged 21 to 50 years and about 30% of subjects > 50 years of age have severe periodontitis. Importantly, based on cross-sectional and prospective epidemiological studies, periodontitis has been linked to cardiovascular diseases and cerebrovascular ischemia, although mechanisms responsible for this association are obscure.[1]

Several parameters of systemic inflammation have been identified, including levels of C-reactive protein (CRP) in the range 1 to 3 mg/L, and have recently gained special attention as risk factors for cardiac and cerebrovascular events. Also, elevated plasma levels of interleukin-6 (IL-6) have been associated with unstable angina and cardiovascular diseases, and IL-6 is related to other cardiovascular risk factors. Moreover, it has been established that IL-6 induces CRP production.

More recent evidence, however, has indicated that patients with severe periodontitis have increased serum levels of CRP, hyperfibrinogenemia, moderate leukocytosis, as well as increased serum levels of IL-1 and IL-6 when compared with unaffected control populations. These data have received considerable attention, but fall short of indicating that periodontitis was the cause for the observed serum acute phase responses, establishing that periodontitis plays a role in the etiological pathway of systemic inflammatory diseases such as atherosclerosis.[2]

The acute-phase response is a nonspecific process that may occur in the initial host response to injuries, infections, ischemic necrosis, or malignancy. It is initiated by the activation of local macrophages and other cells (including fibroblasts and endothelial cells), leading to the release of mediators such as TNF-α, IL-6, and IL-1β. These, in turn, cause systemic changes including hepatic release of a range of plasma proteins (including CRP), activation of complement proteins, and various metabolic changes. IL-6 also promotes the induction of fibrinogen, haptogloblin, α1-antitrypsin, and α2-macrogloblin among others. CRP and other acute-phase molecules are usually present at relatively low levels in plasma, but may be raised dramatically within 72 h of tissue injury, or with infection. CRP opsonizes bacteria for complement-binding and activates complement when complexed. CRP, IL-1β, IL-6, and TNF-α have been associated with the presence of various bacterial infections, including periodontitis.[3]

CRP levels appear after the onset of disease and levels increase within 4-6 h after an acute tissue injury, whereas serum levels of all the other acute phase reactants increase 12-24 h from injury. CRP is consistently found in bacterial infection, acute rheumatic fever, and malignant diseases, viral infections, tuberculosis, and also in patients following surgical operations and blood transfusions.[4]

Numerous cytokines have been identified at sites of chronic inflammation such as arthritis and periodontitis. One of these, interleukin-6 (IL-6), is a major mediator of host response to tissue injury and infection. IL-6 plays a major role in B cell differentiation and also enhances T cell proliferation and bone resorption. There is a significant correlation between tissue levels of IL-6 and the severity of the coincident inflammation. However, the role of IL-6 in the etiology of periodontal disease remains unclear and its role in the resorption of alveolar bone coincident to periodontitis has not been specifically determined.[5]

Spontaneous production of IL-6 has been reported in mononuclear cells isolated from inflamed gingival tissues of patients with periodontitis. IL-6 levels may correlate with the severity of periodontal disease. IL-6 is elevated in sites of refractory periodontitis compared to sites of stable, advanced periodontitis, which suggests that it could be a diagnostic marker for sites of active periodontal disease.[5]

There is increasing evidence that chronic infections as well as inflammatory mechanisms play a major role in atherogenesis and cardiovascular diseases (CVD). Several studies suggested an association between periodontal diseases and atherosclerosis. Direct and indirect host-mediated effects of infectious agents may be responsible for the association between infections in general, and periodontitis specifically, and atherosclerosis.[6]

Due to the potential association between periodontitis and cardiovascular disease, CRP and IL-6 have been identified as risk factors for cardiovascular disease. The present study was undertaken to examine whether serum levels of CRP and IL-6 are increased in periodontitis, and to determine the relationship of their elevated levels to the severity of periodontal disease.

### Aims and objectives

To evaluate the levels of C-reactive protein and interleukin-6 in patients with chronic generalized and localized periodontitis when compared to periodontally healthy individuals.

## MATERIALS AND METHODS

Patients for the study were selected from the Department of Periodontics, Meenakshi Ammal Dental College and Hospital. A total number of 28 patients above 30 years of age diagnosed as having chronic periodontitis with a probing pocket depth > 5 mm and radiographic evidence of bone loss were included in the study. Fourteen periodontally healthy individuals in the same age group were included as controls.

### Exclusion criteria

1. Smoking
2. Pregnant women
3. Individuals with acute or chronic medical disorders including diabetes, viral, fungal, or bacterial infections
4. Trauma and recent tooth extractions
5. Patients with < 27 teeth

Forty-two patients were equally divided into three groups based on their clinical attachment level (CAL).

#### Group I: (Controls)

Free from periodontal disease.

#### Group II:

Chronic localized periodontitis.

#### Group III:

Chronic generalized periodontitis.

The clinical periodontal parameters used were the Gingival Index (Loe and Silness, 1963) and Clinical Attachment Level (CAL) [Tables 1 and 2].

**Table 1:** Gingival index scores in different study groups

Sl. No	Group I	Group II	Group III
1	1.2	1.3	2.33
2	1.22	1.4	2.12
3	1.2	1.4	2.12
4	1.34	1.4	2.25
5	1.22	1.55	2.09
6	1.29	1.7	2.14
7	1.16	1.8	2.04
8	1.36	1.8	2.06
9	1.25	1.3	2.16
10	1.17	1.5	1.95
11	1.36	1.4	2.18
12	1.25	1.4	2.12
13	1.28	1.5	2.2
14	1.33	1.3	2.25
Mean ± S.D	1.26 ± 0.07	1.48 ± 0.17	2.14 ± 0.10

Inference: Mean gingival index score in group III (2.14 ± 0.10) was higher than the mean gingival index scores in group II (1.48 ± 0.17) and in group I (1.26 ± 0.07)

**Table 2:** Gingival index parameters for the different study groups

Group	Mean ± SD	*P* value	Significant groups at 5% level
I	1.26 ± 0.07	< 0.0001 (Sig)	III *Vs* I, II
II	1.48 ± 0.17		II *Vs* I
III	2.14 ± 0.10		

Inference: Mean gingival index score in group I was 1.26 ± 0.07, in group II was 1.48 ± 0.17 and in group III was 2.14 ± 0.10. Mean gingival index scores were statistically significant when group III was compared to group I and group II. Mean gingival index scores were also statistically significant when group II and group I were compared

Criteria followed for Diagnosing Periodontitis. (1999, AAP International workshop)

1. Patients with >30% of sites with loss of attachment were classified as having generalized periodontitis.
2. Patients with <30% of sites with attachment loss were classified as having localized periodontitis.

### Method of collection of serum

Venous blood was collected from each subject in ethylenediamine tetraacetic acid (EDTA)-containing tubes. Tubes were immediately put on ice, centrifuged, and serum isolated within two hours. Serum samples were stored in a storage vial box containing a dry ice pack, before being transferred and stored in a freezer at –70°C.

### Estimation of CRP

Serum levels of CRP were determined using the particle-enhanced turbidimetric immunoassay (PETIA technique. The reference range in this method is 0.2-0.3 mg/dL [Tables 3 and 4].

**Table 3:** Serum C-reactive protein levels (mg/dL) in different study groups

Sl. No	Group I	Group II	Group III
1	0.34	0.21	0.69
2	0.29	0.42	0.23
3	0.25	1.31	0.24
4	0.33	0.99	0.23
5	0.29	0.21	0.75
6	0.20	0.54	0.19
7	0.30	0.83	1.07
8	0.39	0.3	0.56
9	0.29	0.36	0.33
10	0.25	0.19	0.58
11	0.48	0.23	0.59
12	0.34	0.35	0.64
13	0.31	0.24	0.71
14	0.20	0.52	0.80
Mean ± SD	0.304 ± 0.073	0.479 ± 0.339	0.544 ± 0.264

Inference: Mean value of C-relative proteion in group I was 0.304 ± 0.073 mg/ dL, Group II was 0.479 ± 0.339 mg/dL, and group III was 0.544 ± 0.264 mg/dL. Mean value of CRP in group III was higher than group II and group I means

**Table 4:** C-reactive protein parameters for different study groups

Parameter	Group	Mean ± SD	*P* value*	Significant# group at 5% level
	I	0.304 ± 0.073		
CRP	II	0.479 ± 0.339	0.04 (Sig)	III *Vs* I
	III	0.544 ± 0.264		

Inference: Mean C-relative proteion value in group I was 0.304 ± 0.073, in group II was 0.479 ± 0.339 and in group III was 0.544 ± 0.264. When group III and group I were compared, the difference in the CRP levels was statistically significant (*P* < 0.05), however no other differences were statistically significant (*P* > 0.05)

### Principle of procedure

In this method, latex particles were coated with an antibody directed against the C-reactive protein aggregate in the presence of C-reactive protein in the sample. The increase in turbidity which accompanies aggregation is proportional to the C-reactive protein concentration.

### Estimation of IL-6

Serum levels of IL-6 were determined by using the chemiluminescent immunoassay. Reference range in this method is nondetectable to 5.9 pg/mL.

### Principle of procedure

The IL-6 test unit contains one bead coated with a monoclonal, murine anti-IL-6 antibody that aggregates in the presence of IL-6 in the sample.

## RESULTS

A total of 42 individuals, both males and females above the age of 30 years, were included in this study.

A single examiner conducted the periodontal assessment in order to minimize the variation in the data.

**Figure 1:**
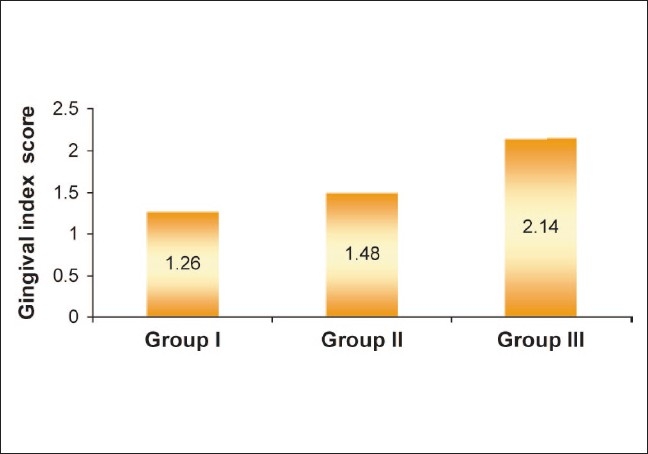
Mean gingival index scores in different study groups

**Figure 2:**
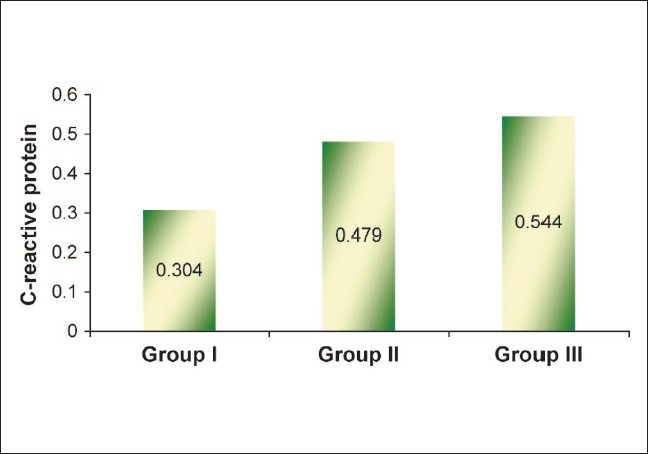
Mean values of C-reactive protein in different study groups

All the serum samples of CRP were estimated by using the particle-enhanced turbidimetric immunoassay (PETIA) technique whereas IL-6 levels were estimated by using the chemiluminescent (IMMULITE) technique [Figures 1 and 2].

### Statistical analysis

Means and standard deviations were estimated from the samples for each study group. Mean values were compared by using one-way analysis of variance (ANOVA). Multiple range tests according to the Tukey-honestly significant differences (HSD) procedure were employed to identify the significant groups at 5% level. Medians and ranges were estimated for certain parameters wherever appropriate. Median values were compared between different study groups by the median test (nonparametric procedure). In the present study, *P* < 0.05 was considered as the level of significance.

## DISCUSSION

Epidemiological studies have implicated periodontitis as a risk factor for the development of cardiovascular disease (CVD). Persistent infections such as periodontitis induce inflammatory and immune responses which may contribute to coronary atherogenesis, and may lead to coronary heart disease (CHD) in conjunction with other risk factors.

Cardiovascular diseases are a heterogenous group of conditions that cause significant morbidity and mortality. During the last decade, poor dental health has been found to be significantly correlated to cardiovascular disease,[7,8] and a significant association between periodontal disease and coronary heart disease was reported by Beck *et al*.[9]

Studies by Ridker[10] showed evidence for inflammation playing a role in the pathogenesis of cardiovascular disease. Some inflammatory or hemostatic markers constitute cardiovascular risk factors. In this respect, the acute phase reactant, C-reactive protein, is of special interest. CRP represents an emerging and reliable marker of the acute phase response to infectious burdens and/or inflammation. As a consequence of its kinetics, it best describes the inflammatory status of the individual.[11]

CRP hepatic production is usually elicited by an inflammatory stimulus and mediated through a complex network of cytokines, mainly IL-6.[12] CRP has also assumed a significant role as a predictor for future coronary events in healthy populations.[13]

Systemic low-grade infections with their moderate acute phase responses may accelerate the formation of atheromatous plaques with a consequent increased risk of future cardiovascular events.[14]

Chronic infections which are known to cause a rise in circulating CRP levels also lead to a higher risk for cardiovascular disease. Periodontal disease being a chronic infection, shares pathogenic mechanisms of cardiovascular diseases with the release of some inflammatory mediators like PGE2, IL-1, IL-6, and TNF-α. These mediators can also initiate a systemic acute phase response.

IL-6 is an inflammatory cytokine that is released from monocytes, lymphocytes, or endothelial cells at sites of tissue injury. IL-6 stimulates the release of neutrophils and platelets from the bone marrow into circulating blood, a part of acute and chronic inflammatory reactions. IL-6 and other cytokines alter hepatic protein synthesis, increasing the synthesis of C-reactive protein (CRP), serum amyloid A (SAA), fibrinogen, and other hemostatic variables, and decreasing the synthesis of albumin, lipoproteins, and other “negative reactant proteins”.

The elevated levels of CRP and IL-6 in periodontitis patients may occur when bacteria and bacterial products, such as lipopolysaccharide (LPS), as well as locally produced pro-inflammatory cytokines enter the circulation. CRP and IL-6 may contribute, in part, to the observed associations between chronic infections and cardiovascular diseases. CRP may activate complement in damaged vessel walls whereas IL-6 has pro-inflammatory properties and a procoagulant effect. These properties may contribute to the pathogenesis of coronary syndromes. Furthermore, IL-6 stimulates the production of CRP by hepatocytes.

The present study was therefore undertaken to evaluate cardiovascular risk in patients with chronic generalized and localized periodontitis by evaluating their serum CRP and IL-6 levels, comparing them with those of controls.

In this study, the mean level of CRP in the chronic generalized periodontitis group was 0.544 mg/dL, 0.479 mg/dL in the chronic localized periodontitis group, and 0.304 mg/dL in the control group. Comparison among these three groups showed a statistically significant increase in mean CRP levels in chronic generalized periodontitis patients. This is in accordance with the findings of studies done by Loos *et al*.,[1] Slade *et al*.,[4] Fredriksson *et al*.,[15] and Noack *et al*.[16]

Haraszthy *et al*.[17] have given cause for suspecting a correlation between periodontal disease and cardiovascular disease, and atherosclerotic plaques have been shown to contain serotypically identical periodontopathogens from the oral cavity. CRP has been used as a marker for atherosclerotic cardiovascular patients and its level has been used to identify risk in such patients as well.

The normal range of CRP levels in healthy individuals was noted to be 0.2-0.3 mg/dL. CR*P* > 0.3 mg/dL is considered indicative of high risk for developing atherosclerotic cardiovascular disease. It is interesting to note that in the present study, CRP levels in both test groups indicated that patients with either chronic generalized or chronic localized periodontitis may be at higher risk for atherosclerotic cardiovascular disease.

The control group demonstrated a near-normal CRP level (0.304 mg/dL), indicating little or no risk for CVD. While this difference between the control and the chronic localized periodontitis group was not statistically significant, the elevated CRP levels in both the test groups above the margin of risk cannot be ignored. CRP is a very early and sensitive marker of chronic inflammation. In a systemic environment that does not cloud its levels, its value can yield very useful information for the diagnosis of the disease.

As IL-6 is a systemic marker of cardiovascular diseases, this was also used in this study to evaluate its relationship between periodontitis and cardiovascular diseases.

The median levels of IL-6 were 6.35 pg/mL in the chronic generalized periodontitis group and < 5.0 pg/mL in both the chronic localized periodontitis and control groups. The median level of IL-6 in the chronic generalized periodontitis group was higher than in the other groups, but this difference was not statistically significant. In studies done by Loos *et al*.,[1] Anila Prabhu *et al*.,[18] Ebersole *et al*.,[19] and Ebersole and Cappelli,[20] it was found that periodontitis patients have higher CRP and IL-6 levels when compared to the periodontally healthy population.

However, Ide *et al*.[3] reported that there were no statistically significant changes in the levels of any of the aforementioned systemic markers. They concluded that improvement in periodontal health also did not influence the levels of vascular markers.

When serum CRP and IL-6 levels were compared between different groups, a statistically significant correlation was seen between these levels and disease in the chronic localized periodontitis group alone. However, Loos *et al*.[1] had shown that there was a significant correlation between these levels and disease in their chronic generalized periodontitis group also when compared with the other groups.

Comparison between CRP and total counts showed that there was no statistically significant correlation between any of the three groups. This is in contrast with studies reported by Loos *et al*.[1] who found a statistically significant correlation.

There was a statistically significant correlation between CRP levels and the neutrophil count in the chronic generalized periodontitis group alone, rather than the other two groups. This is in agreement with the findings of the study conducted by Loos *et al*.

When IL-6 levels and total leukocyte counts were correlated, there was no significant correlation among the groups. This is in contrast with studies reported by Loos *et al*. Who found statistically significant correlation in the chronic generalized periodontitis group.

Moreover when IL-6 levels and neutrophil count were correlated, there was no correlation seen between the three groups. This is in contrast with the studies conducted by Loos *et al*. who found a statistically significant correlation.

Periodontitis is a mixed infection of supporting structures of the teeth, and thus leads to a port of entry for bacteria, resulting in bacteremia. The total volume of inflamed periodontal tissue may also play a vital role. It has been established that the extent of bacteremia is directly related to the severity of periodontal inflammation. Subsequently, the bacteremia as well as cytokines from the periodontal lesion will stimulate the hepatocytes and leucocytes to produce CRP and IL-6. In the present study, there was a tendency for higher CRP levels in chronic generalized periodontitis compared to chronic localized periodontitis.

It may be speculated that periodontitis may predispose affected patients to cardiovascular diseases by increasing the levels of acute phase proteins which may lead to increased inflammatory activity in atherosclerotic lesions.

The quest for markers that are indicative of periodontal disease, is a long one, and the correlation of marker levels with systemic disease multiplies the importance of such makers.

## CONCLUSION

The relationship between periodontal disease and cardiovascular disease is a scientifically established interplay that has its basis in altered immunologic mechanisms. The ability to predict cardiovascular risk markers in periodontal disease would therefore have immense value in the prevention and treatment of such disease.

Thus, it may be concluded from the present study that C-reactive protein and IL-6 have potential utility as risk markers for cardiovascular disease in the study population. Further research focusing on a large sample size is needed to establish these findings.
